# Degloving injuries with versus without underlying fracture in a sub-Saharan African tertiary hospital: a prospective observational study

**DOI:** 10.1186/s13018-017-0706-9

**Published:** 2018-01-05

**Authors:** Hervé Monka Lekuya, Rose Alenyo, Isaac Kajja, Alexander Bangirana, Ronald Mbiine, Ater Ngoth Deng, Moses Galukande

**Affiliations:** 1 Department of Surgery, Makerere University College of Health Sciences, Kampala, Uganda; 2 Department of Orthopedics, Makerere University College of Health Sciences, Kampala, Uganda; 3 Accident and Emergency Department, Mulago National Referral Hospital, Kampala, Uganda

**Keywords:** Degloving, Soft tissue injury, Morel-Lavallé lesion, ISS, Trauma, Fracture, Debridement, Infection

## Abstract

**Background:**

Degloving injuries are surgical conditions in which an extensive portion of skin and subcutaneous tissue is detached from the underlying fasciae, muscles, or bone surface. Frequently, there is an association of fracture underlying the degloved area. We aimed to compare the short-term outcomes of degloving injuries with and without underlying fracture.

**Methods:**

A prospective cohort study was conducted. We recruited patients with degloving injuries, and followed them up for 30 days to assess the outcomes. We collected data on socio-demography, cause and mechanism of injury, presence of underlying fracture, presence of shock at admission, injury severity score, location and size of degloving injuries, their management, and short-term outcomes. There were two comparison groups of degloving injuries based on the presence or absence of underlying fracture. We analyzed the differences between the two groups by using Fisher exact test for categorical variables and Student’s *t* test for continuous variables; *p* values < 0.05 were considered to be significant. Risk ratio was calculated for the short-term outcomes.

**Results:**

There were 1.56% (*n* = 51) of degloving injuries among 3279 admitted trauma patients during the study period of 5 months; 1% (*n* = 33) with and 0.56% (*n* = 18) without underlying fracture. For the overall degloving injuries, male-female ratio was 2 and mean age was 28.8 years; they were caused by road traffic crashes in 84%, and resulted in shock at admission in 29%. In the group with underlying fracture, lower limbs were frequently affected in 45% (*p* = 0.0018); serial debridement and excision of the avulsed flap were the most performed surgical procedures in 22% (*p* = 0.0373) and 14% (*p* = 0.0425), respectively; this same group had 3.9 times increased risk of developing poor outcomes (mainly infections) after 30 days and longer hospital stay (26.52 ± 31.31 days, *p* = 0.0472).

**Conclusion:**

Degloving injuries with underlying fracture are frequent in the lower limbs, and have increased risk of poor short-term outcomes and longer hospital stay. We recommend an early plastic surgery review at admission of patients with degloving injuries with underlying fracture to improve the flap viability and reduce the infection risk.

**Electronic supplementary material:**

The online version of this article (10.1186/s13018-017-0706-9) contains supplementary material, which is available to authorized users.

## Background

Degloving injury or degloving soft tissue injury has been defined as an avulsion of soft tissue, in which an extensive portion of skin and subcutaneous tissue is detached from the underlying fascia, muscles, or bone surface [1]. These injuries are secondary to shearing forces applied to the tissues, as they happen in road traffic crashes (RTC) [2]. The first reports date back to the early twentieth century, in upper limb injuries caused by occupational accidents with drying machines in laundries, known in the literature as wringer arm [3]. With the advent of the automobile industry, the most frequent trauma mechanism became trampling [2, 4–6]. There are often high energy involved, involving heavy vehicles with little protection as the case of motorcyclists [2]. This new trend is associated with underlying fractures in 40–85% [2, 4, 7]. They occur as monotrauma or as polytrauma with or without massive blood loss; this has a significance in the estimation of injury severity and the requirement of additional therapeutic options [8, 9]. In degloving injuries, the musculo-cutaneous perforators are ruptured but the skin cover is often viable. Sometimes, traumatic shearing force or crush injury acting on the skin surface can cause a separation of the intact skin and subcutaneous tissue from the underlying fascia; it will create a cavity filled with hematoma and liquefied fat. It commonly occurs over the greater trochanter, but may also occur in the flank and lumbo-dorsal region; this feature is referred to as closed internal degloving, [4, 5, 9, 10]. Its location over the greater trochanter is known as Morel-Lavallé lesion (MLL), as it was first described in the midst nineteenth century [5].

Degloving injuries are managed based on the viability of soft-tissue and the presence of fracture in the degloving-affected areas [1, 7]. This presence of underlying fracture implies a high energy involvement [11]. It is generally accepted that the golden time for avulsing injury treatment is 8 h after injury, because the avulsed skins gradually develop ischemia and necrosis due to circulation disorder [12]. The straightforward treatment in emergency is debridement and repositioning of the avulsed flap back into their original position [1], but it is reported that necrosis of the repositioned flap occurs frequently due to the flap viability prior reposition [2, 13, 14]. Clinical assessment of the viability of soft-tissue envelopes by direct inspection is a weak predictor of the extent of injury [2]; it remains less accurate and challenging in practice [5, 15]. The use of intravenous fluorescein has been proposed as a better assessment method, but may overestimate the line of demarcation between viable and nonviable skin [16]. Generally, after incomplete avulsion, skin color, skin temperature, pressure reaction, and bleeding patterns should be examined carefully to assess tissue viability [17, 18]. Nevertheless, if there is total excision of the avulsed tissue, it will lead to extensive tissue loss, increased morbidity, need for new donor sites, increased number of surgical procedures, and prolonged of hospital stay with increased cost. In addition, if there is underlying fracture, there may be need of reduction and fixation before the soft tissue definitive treatment [2, 7]. Uganda’s growing population and increased motorization, combined with a diverse mix of road users including pedestrians, bicycles, handcarts, motorcycles, cars, busses, trucks, trailers, and animals make the road environment increasingly complex [19, 20]. These predispose the population to an increased risk of complex trauma. Delineating the types of degloving injuries in relation to underlying fracture is the cornerstone of establishing a standardized protocol of their management. The purpose of this study therefore was to compare the outcomes of degloving injuries with and without underlying fracture in view of improving their management in a resource constrained environment.

## Methods

### Study design and setting

This was a prospective cohort study done in the surgical units of Mulago National Referral Hospital (MNRH), Kampala, Uganda. MNRH is a tertiary care and main teaching hospital of Uganda. Its unit of accident and emergency (A & E) admits and treats a high volume of trauma patients and surgical emergencies from Kampala and all over the country. We recruited trauma patients presenting with degloving injuries at the A & E from 15th of November 2016 to 15th of April 2017 and followed them up in the different surgical units for a period of 30 days.

### Patient selection

We diagnosed degloving injury by clinical inspection of any avulsion of soft-tissue, in which an extensive portion of skin and subcutaneous tissue detached from the underlying fascia and muscles and having at least 5 cm of its small diameter for open degloving injury; for closed degloving injury, accurate diagnosis was made by additional clinical detection of a fluctuant area combined with the findings of most appropriate imaging modalities [8]; basically, ultrasound or computerized tomography scan (CT scan) were considered in this study. In case of underlying fracture, similar criteria of AO/ASIF classification of soft-tissue injury were applied: injury equal or greater than IO/IC 3 was considered as a degloving injury [21]. Adequate photo-documentation of the initial injury was captured to allow accurate diagnosis prior to definitive management. We included consecutively patients who presented with a degloving injury in any surface of the body region within the first 12 h of injury. We excluded patients whose injuries have been managed definitively from another hospital prior referral.

### Study procedure

Advanced trauma life support (ATLS) guidelines were followed in all patients with degloving injuries on arrival at the A & E. The unstable patients were stabilized during the primary survey of ATLS by the admitting trauma team. During the secondary survey of ATLS, the patients or next to kin were interviewed after written informed consent, and the patients’ clinical parameters were also recorded. At the time of wound exposure and assessment, we captured adequate photo-documentations of degloving injuries using a digital camera (Nikon D5300 digital SLR camera); at least two different views were required with adequate focus and lighting on a white background. We also investigated clinically and radiologically potential underlying fractures and associated injuries. We followed up the patients and recorded the definitive management of the degloving injuries; we finally reassessed the degloving wounds 30 days later after the definitive treatment. For patients who were discharged in a shorter period, we recalled them to come at the 30th day after definitive treatment in the outpatient department.

### Study variables

We collected data on socio-demographic characteristics (age, gender, and main occupation), cause of injury, trauma mechanism, presence of shock at admission, presence of underlying fracture, injury severity score (ISS), associated injuries, location of degloving injuries, their classification and degloved body surface (DBS), hospital accessibility, acute phase management, definitive management, short-term outcomes, mortality, and length of hospital stay.

### Data quality control

Patients with systolic blood pressure below 90 mmHg at admission were considered having hemorrhagic shock; for patients below 10 years, corresponding pediatric vital parameters had been applied for each specific range of age. The severity of injury was assessed posteriorly by using the ISS coding system. The ISS was calculated by using a chart based on the parameters of the abbreviated injury score (AIS) coding. The AIS was then obtained after screening clinically and radiologically all the eventual injuries of the six body regions of patients. An ISS coding chart or a computerized online program (in case of complex injury) was used for calculation [22].

The DBS was expressed both in centimeter square (cm^2^) and in percentage. The DBS in cm^2^ was measured by using a sterile wound measuring guide (3M™ Skin Health) to determine the wound diameter and to calculate the surface according to the geometric formula of the approximate shape (circle, triangle, and so forth) of the degloving wound. The DBS in percentage of body surface area was determined by using the Lund and Browder chart [23].

### Statistical analysis

Data were expressed as proportions, medians, or means ± standard deviation (SD) as appropriate. Differences in categorical variables between respective comparison groups were analyzed using chi-square test or Fisher exact test for categorical variables. The risk ratio (RR) and 95% confidence interval were calculated according to Altman (1991). The continuous variables were analyzed using Student’s *t* test. Two-tailed *p* values < 0.05 were considered significant. Data analysis was carried out using the Statistical Package for Social Sciences version 18 (SPSS Inc. Chicago, USA).

### Ethical considerations

This study was approved by the School of Medicine Research and Ethics Committee (SOMREC) of Makerere University College of Health Sciences (MU-CHS), registered as #REC REF 2017-046. Patients (or next to kin) consented for recruitment in the study and for data publication, including their images.

## Results

**Fig. 1:**
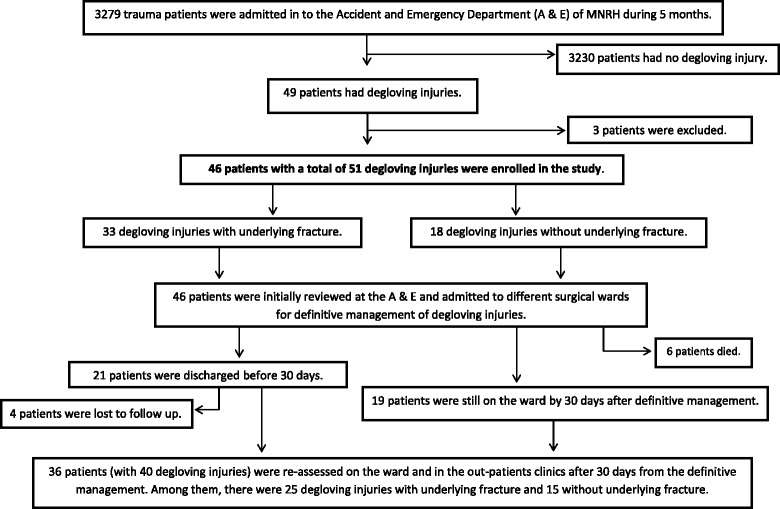
Patients’ flow chart

**Fig. 2:**
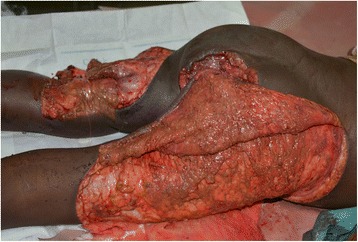
Bilateral degloving injuries of both lower limbs at admission: a 3-year-old male patient who was trampled by a trailer (photo: Lekuya M.H)

There were a total of 3279 trauma patients admitted in A & E during a period of 5 months. Among them, we identified 49 patients who presented with degloving injuries. After excluding 3 patients, we recruited 46 patients with a total of 51 degloving injuries into this study as shown in the patients’ flow chart (Fig. 1). Some patients had multiple location of degloving injuries as illustrated in Fig. 2. The prevalence of overall degloving injuries among trauma patients was 1.56% (*n* = 51); there were 33 (1%) with underlying fracture and 18 (0.56%) without underlying fracture. A total of six patients died and four patients were lost to follow up after discharge.

The majority of the patients were male in 67%; male-female ratio was 2:1. Underlying fractures were more frequent among female patients in 28% with a significant difference (*p* = 0.0487). The mean age was 28.8 ± 12.8 years without significant difference between the two groups (Table 1).

**Table 1:** Socio-demography of patients with degloving injuries and injury related factors

Variables	Frequency (*n*, %)			*p*
	Overall of degloving injuries	Degloving injuries with underlying fracture	Degloving injuries without underlying fracture	
Total of patients	*n* = 46	30 (65.22)	16 (34.78)	–
Total of degloving injuries	*n* = 51	33 (64.71)	18 (35.29)	–
Gender of patients (*n* = 46)				
Male	31 (67.39)	17 (36.96)	14 (30.43)	0.0487
Female	15 (32.61)	13 (28.26)	2 (4.35)	0.0487
Age of patients in years (*n* = 46)				
Mean (±SD)	28.8 (± 12.8)	26.7(± 13.4)	32.7 (± 11.1)	0.1520
Median (range)	27.5 (3–65)	25 (3–65)	33 (10–53)	–
Cause of injury (*n* = 51)				
RTC	43 (84.32)	30 (58.82)	11 (21.56)	0.0228
Car accident as a pedestrian	12 (23.53)	10 (19.61)	2 (3.92)	0.1740
Motorcycle as a passenger	10 (19.61)	6 (11.76)	4 (7.84)	0.7272
Truck/trailer accident as pedestrian.	10 (9.61)	10 (19.61)	0	0.0092
Car accident as a passenger	9 (17.65)	5 (9.80)	4 (7.84)	0.7029
Motorcycle as a pedestrian	2 (3.92)	1 (1.96)	1 (1.96)	1.0000
Machine from industry	3 (5.88)	0	3 (5.88)	0.0392
Assault	2 (3.92)	0	2 (3.92)	0.1200
Unknown causes	3 (5.88)	1 (1.96)	2 (3.92)	1.0000
Trauma mechanism (*n* = 51)				
Knocking or collision	23 (45.10)	13 (25.49)	10 (19.61)	0.3783
Trampling	17 (33.33)	17 (33.33)	0	0.0001
Cutting	4 (7.84)	0	4 (7.84)	0.0122
Ejection from vehicle	3 (5.88)	2 (3.92)	1 (1.96)	1.0000
Others or undetermined	4 (7.84)	1 (1.96)	3 (5.88)	0.1200
Patients presenting with shock (*n* = 46)				
No	34 (73.91)	21 (45.65)	13 (28.26)	0.4977
Yes	12 (26.09)	9 (19.57)	3 (6.52)	0.4977
Injury Severity Score (*n* = 46)				
Mean (±SD)	22.47(± 18.38)	27.18 (± 21.02)	13.83(± 6.25)	0.0117
Median (range)	16 (4–75)	17 (9–75)	13 (4–25)	–

Table 1 shows also that RTC was the leading cause of injury (84%) resulting in an increased number of degloving injuries with underlying fracture (59%) with significant difference (*p* = 0.02). Car accident (as pedestrian) or motorcycle accident (as a passenger) were observed in 63% of degloving injuries.

The most common trauma mechanism was knocking (45%), followed by trampling which lead to degloving injury with underlying fracture in 33% (*p* = 0.0001). The mean ISS was greater in the group of degloving injuries with underlying fracture (13.83 ± 6.25 versus 27.18 ± 21.02; *p* = 0.0117). The ISS above 25 was associated with underlying fracture. There were 29.41% who presented with shock at admission without significant difference between the 2 groups of degloving injuries (*p* = 0.2025).

**Fig. 3:**
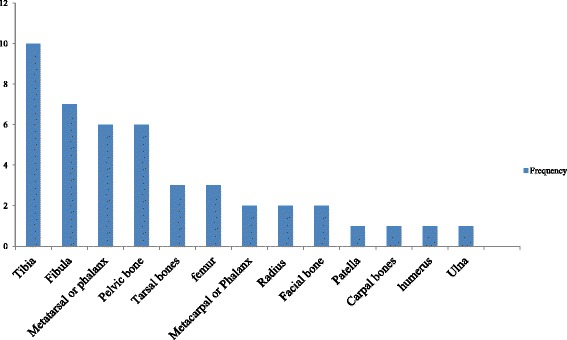
Distribution of underlying fractures among patients with degloving injuries

Underlying fractures of both the tibia (19.6%) and fibula (13.7%) were the most frequent, followed by fracture of foot bones (tarsal, metatarsal, and phalanx) and pelvic bones as shown in Fig. 3.

Table 2 shows that the most frequent anatomical location of degloving injuries was the lower extremity (56.14%) followed by the trunk (19.61%). Degloving injuries of the lower limbs tended to be associated with underlying fracture in 45.10% with a significant difference (*p* = 0.0018), and degloving injuries of the trunk in reverse were not associated with underlying fracture in 13.72% with a significant difference (*p* = 0.0228). About 96% were open degloving injuries. The mean absolute DBS was 476.10 ± 673.72 cm^2^. The median absolute DBS was 204 cm^2^ without significant difference between the 2 groups of comparison (*p* = 0.1591). The mean relative DBS in percentage of total body surface area (TBSA) was 3.27%, with median relative DBS of 1.50% without significant difference (*p* = 0.0777).

**Table 2:** Distribution of anatomical location, classification, and size of degloving injuries

Variables	Frequency (*n*, %)			*p*
	Overall of degloving injuries (*n* = 51)	Degloving injuries with underlying fracture (*n* = 33)	Degloving injuries without underlying fracture (*n* = 18)	
Anatomical location				
Lower limb	29 (56.86)	23 (45.10)	6 (11.76)	0.0018
Trunk	10 (19.61)	3 (5.88)	7 (13.73)	0.0228
Upper limb	8 (15.69)	6 (11.76)	2 (3.92)	0.6959
Head and neck	4 (7.84)	2 (3.92)	2 (3.92)	0.6070
Classification				
Open	49 (96.08)	32 (62.75)	17 (33.33)	1.0000
Closed	2 (3.92)	2 (3.92)	0	0.5341
Degloved body surface (DBS)				
DBS: absolute size (in cm^2^)				
Mean (±SD)	476.10 (± 673.72)	574.69 (± 748.53)	295.33 (± 476.31)	0.1591
Median (range)	204 (50–3200)	250 (80–3200)	148 (50–2100)	–
DBS: relative size (in % of TBSA)				
Mean (±SD)	3.27 (± 3.91)	3.98 (± 4.30)	1.96 (± 2.69)	0.0777
Median (range)	1.50 (0.4–18)	2.00 (0.5–18)	1.00 (0.4–12)	–

**Fig. 4:**
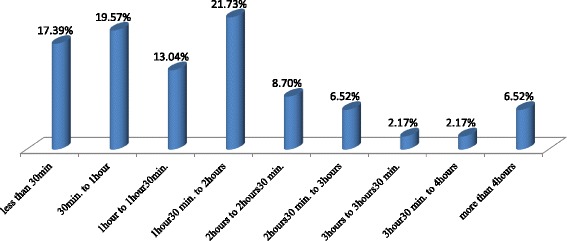
Distribution of overall degloving injuries related to the interval of time between injury and admission

**Fig. 5:**
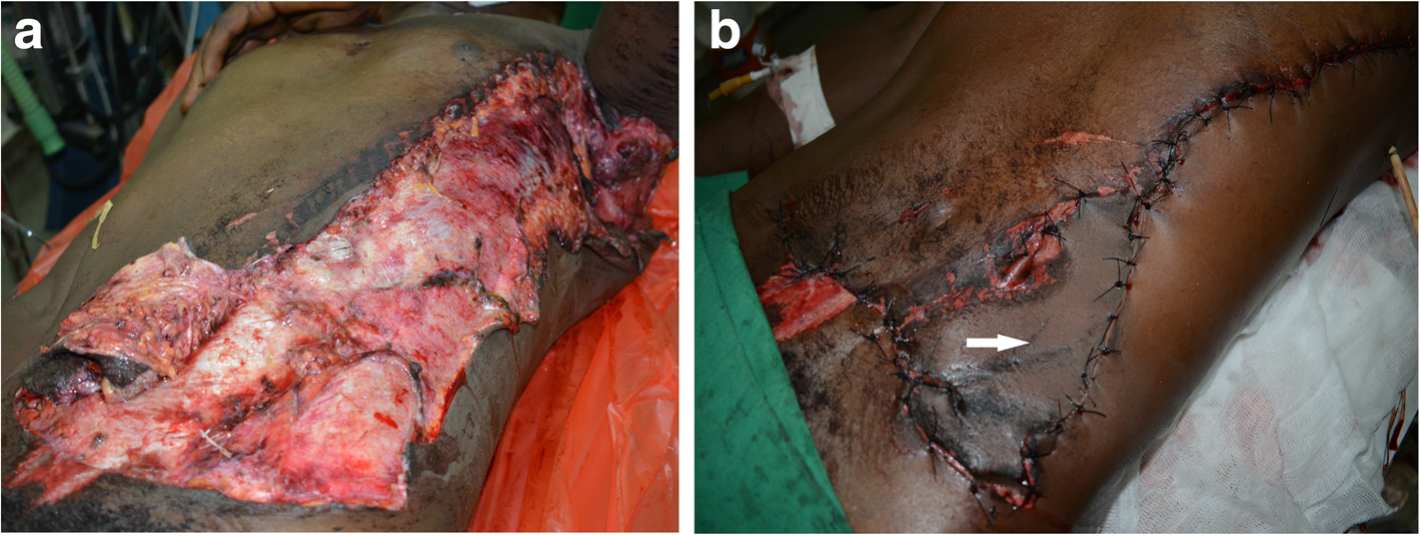
Degloving injury of the trunk: **a** large degloving caused by an industrial cutting machine, **b** Debridement and primary closure after defatting the abdominal degloved skin indicated by a white arrow (photos: Lekuya M.H)

Figure 4 shows that the majority of patients with degloving injuries reached the hospital within 120 min from the injury time (71.73%), and the culminant interval of time is between 90 to 120 min from the injury time (21.73%). Table 3 shows that the majority of patients reached the hospital without any treatment of the wound (60.78%); about 33.33% of patients benefited from wound bandage coverage prior to be transferred to the A & E. The majority received analgesics (98.04%), antibiotics (92.16%), and intravenous fluids (86.27%) at the admission in A & E. Table 3 shows that serial dressing was the most common therapeutic practice of overall degloving injuries, followed by primary debridement and closure of the avulsed flap (respectively 76.47 and 37.25%); Fig. 5 illustrates an example of primary debridement and closure in our setting. Patients received routinely antibiotics and analgesics during the definitive management. There were four patients who benefited from diverting colostomies (7.84%) in the group of degloving injuries with underlying fracture of the hip region; an illustration of a case of unstable pelvic fracture with left thigh MLL in a patient who benefited from a diverting colostomy is available in Additional file 1.

**Table 3:** Management options of degloving injuries

Variables	Frequency (*n*, %)			*p*
	Overall of degloving injuries (*n* = 51)	Degloving injuries with underlying fracture (*n* = 33)	Degloving injuries without underlying fracture (*n* = 18)	
Pre-hospital management				
None	31 (60.78)	18 (35.29)	13 (25.49)	0.2470
Bandage	17 (33.33)	13 (25.49)	4 (7.84)	0.3515
Rising with water	5 (98.04)	3 (5.88)	1 (1.96)	1.0000
Acute phase management in the hospital				
Analgesics	50 (98.04)	32 (62.75)	18 (35.29)	1.0000
Antibiotics	47 (92.16)	30 (58.82)	17 (33.33)	1.0000
Intravenous fluids	44 (86.27)	28 (54.90)	16 (31.37)	1.0000
Dressing	39 (76.47)	25 (49.02)	14 (27.45)	1.0000
Rinsing with water	39 (76.47)	25 (49.02)	14 (27.45)	1.0000
Bandage	35 (68.63)	25 (49.02)	10 (19.61)	0.2070
Tetanus prophylaxis	29 (56.86)	17 (33.33)	12 (23.53)	0.3804
Debridement	29 (56.86)	20 (39.22)	9 (17.65)	0.5590
Limb splinting	18 (35.29)	18 (35.29)	0	˂0.0001
Reposition of the avulsed flap without suturing	16 (31.37)	12 (23.53)	4 (7.84)	0.3583
Blood transfusion	13 (25.49)	11 (21.57)	2 (3.92)	0.1033
Others	16 (31.37)	16 (31.37)	4 (7.84)	0.3583
Definitive management: local treatment				
Serial dressing	39 (76.47)	27 (52.94)	12 (23.53)	0.3036
Primary debridement and closure of the avulsed flap	19 (37.25)	10 (19.61)	9 (17.65)	0.2279
External fixator	15 (29.41)	15 (29.41)	0	0.0004
Serial debridement	12 (23.53)	11 (21.57)	1 (1.96)	0.0373
Delayed skin graft	10 (19.61)	9 (17.65)	1 (1.96)	0.0771
Secondary closure	9 (17.65)	4 (7.84)	5 (9.80)	0.2494
Excision of the avulsed flap	6 (11.76)	7 (13.73)	0	0.0425
Primary amputation	3 (5.88)	3 (5.88)	0	0.5436
Hematoma evacuation	2 (3.92)	1 (1.96)	1 (1.96)	1.0000
Defatting and primary grafting of the avulsed skin	2 (3.92)	0	2 (3.92)	0.1200

The short-term outcomes were good in 62.5% for the overall degloving injuries; there was a significant difference in favor of the degloving injuries without underlying fracture in 32.5% (*p* = 0.0197). The significance was markedly found in primary healing outcome with *p* = 0.018 (Table 4).

**Table 4:** Short-term outcomes of degloving injuries (bivariate analysis)

Variables	Frequency (*n*, %)			RR (95% CI)	*p*
	Overall	Degloving injuries with underlying fracture	Degloving injuries without underlying fracture		
Short-term outcomes of the degloving injuries	*n* = 40	*n* = 25	*n* = 15		
Good outcomes	25 (62.5)	12 (30.00)	13 (32.50)	0.55 (0.35–0.87)	0.0197
Primary healing	16 (40.00)	6 (15.00)	10 (25.00)	0.36 (0.16–0.79)	0.0180
Satisfied skin graft take	6 (15.00)	5 (12.50)	1 (2.50)	3.00 (0.39–23.29)	0.3813
Secondary healing	3 (7.50)	1 (2.50)	2 (5.00)	0.30 (0.03–3.03)	0.5445
Poor outcomes	15 (37.50)	13 (32.50)	2 (5.00)	3.90 (1.02–14.96)	0.0197
Local skin infection	8 (20.00)	6 (15.00)	2 (5.00)	1.80 (0.42–7.80)	0.6857
Persistent ulcer	4 (10.00)	3 (7.50)	1 (2.50)	1.80 (0.21–15.78)	1.0000
Underlying osteomyelitis	4 (10.00)	4 (10.00)	0	5.54 (0.32–96.19)	0.2778
Sepsis	3 (7.50)	5 (12.50)	0	6.77 (0.40–114.4)	0.1372
Unsatisfied skin graft take	3 (7.50)	3 (7.50)	0	4.31 (0.24–78.06)	0.2788
Amputated limb	3 (7.50)	3 (7.50)	0	4.31 (0.24–78.06)	0.2788
Necrosis of the repositioned skin or flap	3 (7.50)	1 (2.50)	2 (5.00)	0.30 (0.03–3.03)	0.5445
Necrotizing fasciitis	1 (2.50)	0	1 (2.50)	0.21 (0.009–4.74)	0.3750
Vital outcomes	*n* = 46	*n* = 30	*n* = 16		
Death	6 (13.04)	5 (10.87)	1 (2.17)	2.67 (0.34–20.91)	0.6489
Lost to follow up	4 (8.70)	3 (6.52)	1 (2.17)	1.6 (0.18–14.16)	1.0000
Length of hospital stay (days)	*n* = 46	*n* = 30	*n* = 16	*t* test	
Mean (±SD)	21.0 ± 27.12	26.52 ± 31.31	10.83 ± 12.2	**−** 2.03 (**−** 31.9 to **−**0.2)	0.0472
Median (range)	10 (0.2-107)	12 (0.2–107)	6 (1–45)	–	–

**Fig. 6:**
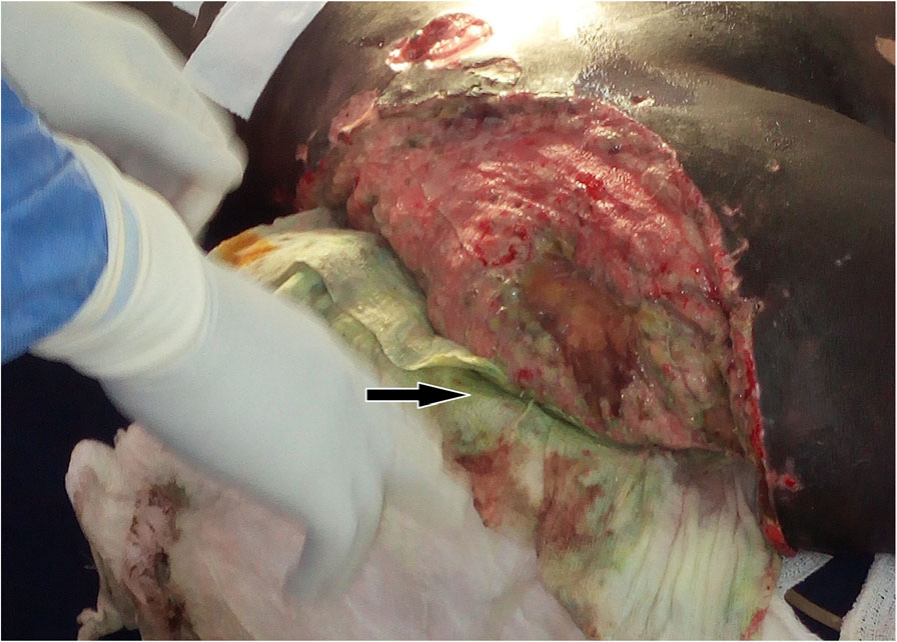
Persistent ulcer with infection at 30 days: dressing change of an infected wound (pseudomonas patterns with black arrow) of an 18-year-old female patient who developed a skin necrosis of a closed degloving injury (photo: Lekuya M.H)

**Fig. 7:**
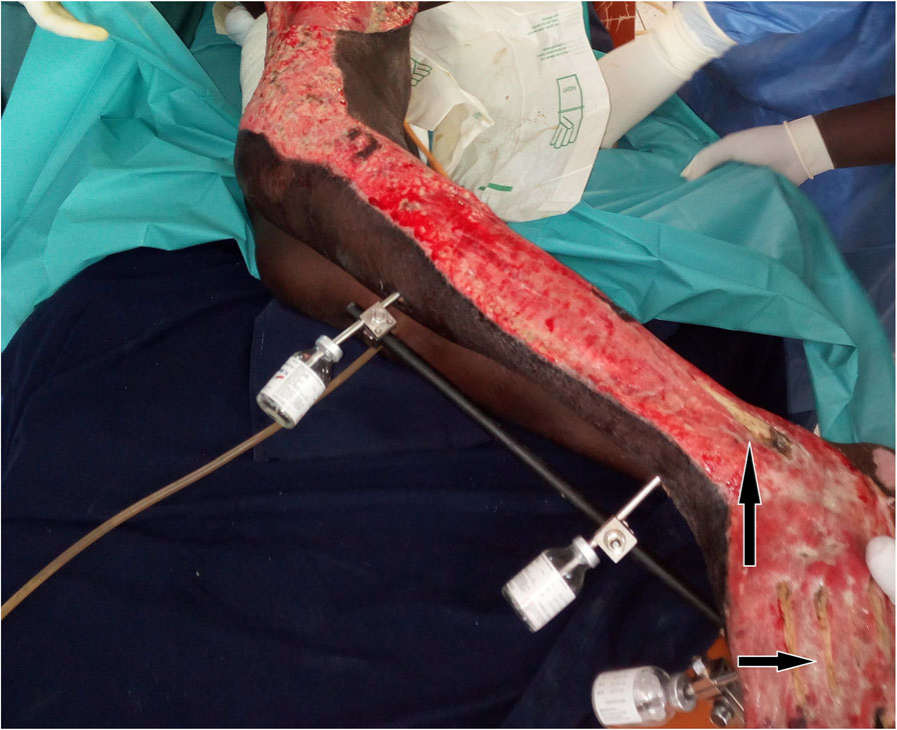
Degloving injury with underlying distal osteomyelitis at 30 days: osteomyelitis of the left ankle and foot (see black arrows) with delayed wound granulation and recurrent infection. The external fixators are in situ (photo: Lekuya M.H)

Degloving injuries with underlying fractures had 3.9 times increased risk of developing poor short-term outcomes (mainly infection) comparing to those without underlying fracture (95% CI 1.02–14.96; *p* = 0.0197; Table 4). Those infection patterns were especially local skin infection, persistent ulcer, osteomyelitis, and sepsis as illustrated in Figs. 6 and 7.

Overall mortality was 13.04%, without significant difference between the compared two groups. The causes of death were hemorrhagic shock (4.35%), severe head injury (4.35%), and sepsis (4.35%). Further mortality analysis shows that patients who arrived at the hospital after 1 h from injury had 2.96 times increased risk of death (95% CI 1.32–6.64; *p* = 0.045). In addition, the risk of death was increased in 6.67 times among patients who presented with hemorrhagic shock at admission (95% CI 3.19–13.94, *p* ˂ 0.0001), and in 5.71 times among patients with the ISS above 24 (95% CI: 2.92–11.20, *p* = 0.0002). There was also a significant difference (*p* = 0.404) between the means of relative DBS (in % of TBSA) of patients who died (4.33%) versus those who survived (2.96%).

The mean of hospital stay in days was 21.0 ± 27.12 days. The degloving injuries with underlying fracture had longer hospital stay (26.52 ± 31.31 days) with significant difference (*p* = 0.0472) as shown in Table 4.

## Discussion

We set out to explore the outcomes of degloving injuries with and without underlying fracture. We reported a prevalence of 1.56% of degloving injuries among trauma patients. Two thirds of those degloving injuries had underlying fracture. This is significant in this setting where there was a high volume of 3479 admitted trauma patients in 5 months without any natural disaster or war.

Degloving injuries occurred more frequently in young males, since they are the most exposed to trauma in everyday life. The overall mean age was 28.8 years; this stands with most of studies where the mean age was between 29 and 31 years [1, 7, 24]. The male-female sex ratio was 2:1; Mello et al. found a similar ratio in Brazil [7]. In our study, females were more frequently affected by degloving injuries with underlying fracture but *p* value was close to 0.05.

Our study found that RTC was the principal cause of overall degloving injuries in 84.32%. This stands with the findings of other studies done on degloving injuries where the RTC-related injury ranges from 45 to 97% [1, 7, 25]. Pedestrians (involved in car accident) or passengers (involved in motorcycle accident) were both frequently observed in RTC-related causes of degloving injuries. We also found that RTC were frequently associated with degloving injuries with underlying fracture (58.82%) with significant difference (*p* = 0.0228). Indeed, the presence of heavy trucks on the roads of Kampala city predisposed the population to degloving injuries with underlying fractures (19.61%, *p* = 0.0092). The most common trauma mechanism of overall degloving injuries was collision or knocking (45.10%), followed by trampling (33.33%). The trampling mechanism resulted significantly to underlying fractures (*p* = 0.0001). Available literatures described causes rather than trauma mechanisms, yet both have shown significance for the determinant of underlying fracture in degloving injuries. Although we emphasize their impact separately in the occurrence of underlying fracture, those trauma mechanisms may occur concurrently during RTC.

The mean ISS was 22.47 ± 18.38 in overall degloving injuries. The mean ISS was greater in the group of degloving injuries with underlying fracture in comparison to the group without fracture (27.18 ± 21.02 versus 13.83 ± 6.25, *p* = 0.0117). In contrast, Hakim et al. in Qatar found that the mean ISS was 13.80 ± 10.9 for degloving injuries [24]. These can be explained by the same fact that our patients sustained high energy trauma (knocking and trampling) with associated injuries. The ISS above 25 was associated exclusively associated with underlying fracture in our study.

About 29.41% of degloving injuries resulted in hemorrhagic shock without significant difference regarding to the presence or absence of underlying fracture; degloving injuries are known to be associated with severe concomitant injuries and massive blood loss [8]. The location of degloving injuries such as the scalp, upper limb, and heel may cause significant blood loss with hemorrhagic shock [14, 15, 26]. Yu Chen et al. found that 29.63% of patients with degloving injuries had hemorrhagic shock at admission. Milcheski et al. in Brazil found 9.5% patients with hemodynamic instability at admission; the contrast of their lower percentage may be explained by their inclusion criteria of unstable patients with several parameters like multiple trauma, multiple transfusions, and hypothermia [13]. In our study, we considered only low blood pressure values at the admission as probable result of severe hemorrhage.

The degloving injuries with underlying fracture were 64.71%. The most frequent underlying fractures were the tibia (19.6%) and fibula (13.73%). Mello et al. found 70% of patients with degloving injuries presented with associated fractures, but they included also fractures of the non-degloving areas. Lower limbs (56.14%) followed by the trunk (21.57%) were the most frequent location of degloving injuries as found also in most of studies on degloving injuries of the limbs and trunk but ranging from 44 to 96% [8, 19, 24, 26]. The primary explanation of this frequency is that the lower limbs and trunk represent more than 70% of the TBSA in general. Degloving injuries of the lower limbs tended to be associated with underlying fracture in 50.98% with significant difference (*p* = 0.0023), and degloving injuries of the trunk in reverse were not associated with underlying fracture in 13.72% (*p* = 0.0369). This can be explained by the position of the patient during the injury impact.

The mean of DBS was 1.50%, ranging from 0.4 to 18% of TBSA and from 50 to 3200 cm^2^ in absolute size. There was no significant difference of DBS regarding the presence or absence of underlying fracture. Mello et al. in Brazil found an average DBS of 8.2 ± 4.5% with a range of 3–22% [7]. Yu Chen et al. found a range of 150–1500cm^2^ in absolute size [12]. Those differences could be resulting in random error due to variation on wound measurement techniques.

There were almost 96.08% of open degloving injuries; Khan et al. found a similar result of 94% of open degloving injuries; in contrast, Hakim et al. found a lower frequency of 79.78%; this could be explained by the fact that they excluded degloving injuries in which the skin was completely detached.

About 71.73% of patients with degloving injuries reached the hospital within 120 min from the injury time. About 6.52% reached the hospital after 4 h from the time of injury. In fact, most of the injuries secondary to RTC occurred in the highway of Kampala city, and the patient’s evacuation should have passed through a traffic jam. In China, Yu Chen et al. reported in their study that patients reached the hospital within 3.5 h (range 1.5–10 h) after injury [12].

Serial dressing was the most common surgical definitive management (76.47%), followed by primary debridement and closure of the avulsed flap (37.25%) for the overall degloving injuries.

In case of presence of underlying fracture, serial debridement and excision of the avulsed flap were commonly performed with significant difference (*p* = 0.0373 and 0.0425). This frequent excision of avulsed flap was a potential loss of tissue; it assumed there was poor assessment of flap viability; this excised flap could have still worked temporarily as a biological dressing. Kudsk et al. suggested that local treatments of degloving injuries should consist of evaluating the viability of the flaps, debridement of necrotic tissues, and use of nonviable flap areas as donor of skin grafts in partial full or thickness [2].

Delayed skin graft was done in 19.61% of overall degloving injuries, but most of them were in the group with underlying fracture (17.65%, *p* = 0.0771). Arnez et al. did 16.5% of skin graft in their series [1]. In most of the studies, delayed skin graft was performed after some days of vacuum-assisted closure (VAC) [1, 12, 13], which was not available for patients in general wards in MNRH during the study period.

There were 5.88% of primary amputations of the limbs. Milcheski et al. reported 28.6% of amputation in their series in Brazil. This high rate seems to be related to their inclusion criteria (only lower limbs) and also can be explained by secondary complications of massive loss of soft tissue [13].

Good short-term outcomes (primary healing, satisfied skin graft take, secondary healing) were observed for the overall degloving injuries in 62.5%; but when comparing the 2 groups, there was a significant difference in favor of the degloving injuries without underlying fracture in 32.50% (*p* = 0.0197). This significance of good outcomes was markedly found in primary healing which was frequent in absence of underlying fracture (*p* = 0.018). Indeed, 6 out of 10 of overall skin grafts were declared satisfied, yet there was no use of VAC (not available) prior skin grafting as described in many studies [1, 12, 13]. In contrast, there was 3.9 times increased risk of developing poor short-term outcomes in the group with underlying fractures (*p* = 0.0197) (Table 4). Lafiti et al. reported that complications of degloving injuries depend on their mechanism, anatomic region, and type and concomitant injuries [8]. In our study, this increased risk could be explained by high energy tissue damage responsible of fractures, and also accessory manipulations by the orthopedic team with meticulous debridement of the soft tissues before closure.

Mortality was 13.04%, without significant difference between the compared two groups. This is basically an in-hospital mortality of degloving injuries; less probably, more severe polytrauma patients with pelvic fracture would have reached the hospital within 2 h of injury. The reported causes of death in the hospital were hemorrhagic shock (4.35%), severe head injury (4.35%), and sepsis (4.35%). Hakim et al. observed an overall mortality of 9.0% (*n* = 16) among patients with degloving injuries, and around half (*n* = 7) of them died within the first 24 h of admission due to severe associated injuries [24]. In our study, we also found that patients admitted after 1 h from the injury time and patients with hemorrhagic shock had increased risk ratio of death, respectively, RR = 2.96 (95% CI 1.32–6.64; *p* = 0.045) and RR = 6.67 (95% CI 3.19–13.94, *p* ˂ 0.0001). Those are commonly reported causes of death of trauma patients in our setting. Indeed, ISS above 24 was found to be associated with mortality of degloving injuries in our study (RR 5.71; 95% CI 2.92–11.20, *p* = 0.0002). Although there is new accurate anatomical score (NISS) to predict mortality in trauma [27], ISS can still be used in degloving injuries to predict mortality. Hakim et al. found that ISS was a mortality predictor of degloving injuries in closed types only (odd ratio 1.2; 95% CI 1.06–1.35; *p* = 0.004); however, this study was a retrospective series [24]. Larger sizes of the degloving wounds have been shown to be associated with mortality in our study; this could be explained by the bleeding patterns and by the increased risk of infections.

The mean of hospital stay was 21.0 ± 27.12 days (range 0.2–107 days) for the overall injuries. Degloving injuries with underlying fracture had longer hospital stay (26.52 ± 31.31 days) with significant difference (*p* = 0.0472). In fact, fracture by itself may lead to increased hospital length of stay in regard to eventual manipulations of the wound. Most of the studies gave an average of hospital stay similar to our findings for the overall degloving injuries; Hakim et al. found in their study a hospital length of stay of days 10 (range 1–393) [8] while Mello reported a mean hospital stay of 47.3 ± 40 days (range 7–239) [7]; Milcheski et al. reported a mean hospital stay of 46.2 days for patients undergoing primary suture and 32.5 days for patients undergoing primary grafting (*p* < 0.001) [13].

### Study limitations

**Fig. 8:**
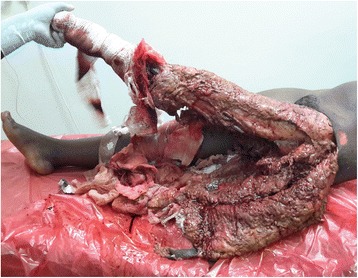
Degloving injury of the entire left lower limb with mangled foot at admission: a 24-years-old female patient who was trampled by a truck (photo: Lekuya M.H)

We analyzed the results into two groups based on the presence or absence of underlying fractures; as a matter of fact, we included complex degloving injuries with mangled extremities combined with intact bones that we could not accurately classify those borderline underlying fractures as illustrated in Fig. 8. The extent and severity of those injuries varied widely, so there might be an extensive degloving injury without underlying fractures, as well as a small degloving injury with underlying fracture (Additional files 2, 3, and 4); this has made the comparative analysis difficult to generalize our findings regarding the size and the severity of degloving injuries. Furthermore, we conducted this study in a single center which has limited resources, and our sample size was fairly small.

## Conclusion

The overall degloving injuries occur more frequently in young males and caused by RTC, by collision and trampling mechanism. Severity of injury increases the presence of underlying fracture. The lower limb is the frequent anatomical location of degloving injuries, and associated with eventual underlying fracture. Serial debridement and excision of avulsed flaps were the most performed surgical treatments of degloving injuries with underlying fracture in MNRH. The risk of poor short-term outcomes (infections) and longer hospital stay is increased with the presence of underlying fractures. Our study did not find an association of underlying fracture with the in-mortality among patients with degloving injuries. However, factors such as hospital admission more than 1 h from the injury, presence of hemorrhagic shock at admission, ISS above 24, and larger DBS were significantly associated with the mortality of degloving injuries.

### Recommendations

We recommend a plastic surgery review at the time of admission of degloving injuries with underlying fracture to improve the flap viability; this would promote early soft tissue reconstruction, improve the outcomes and reduce the risk of infection and hospital stay of degloving injuries. We also advocate for more studies on degloving injuries with complex fractures and long-term outcomes.

## Additional files

Additional file 1: Management of MLL in a 25 years old female patient knocked by a truck: a. X-rays film of an unstable pelvic fracture with a left thigh MLL; b. External fixation, diverting colostomy, hematoma evacuation and compression bandage of the MLL. (JPEG 421 kb)

Additional file 2: Large degloving injury of the entire right upper limb of a 22 years old male patient; a construction worker who was trampled by a trailer. (JPEG 2499 kb)

Additional file 3: Small degloving injury of the dorsum of the left hand. (JPEG 2722 kb)

Additional file 4: Small degloving injury of the dorsum of the left foot. (JPEG 1363 kb)

## Abbreviations

A & E: Accident and emergency unit
DBS: Degloved body surface
ISS: Injury severity score
MLL: Morel-Lavallé lesion
MNRH: Mulago National Referral Hospital
RTC: Road traffic crashes
TBSA: Total body surface area
