# Supplementary figures and images for: Degloving injuries with versus without underlying fracture in a sub-Saharan African tertiary hospital: a prospective observational study
Source: J Orthop Surg Res. 2018 Jan 5;13:2. doi: 10.1186/s13018-017-0706-9 (PMC5756448; doi:10.1186/s13018-017-0706-9)

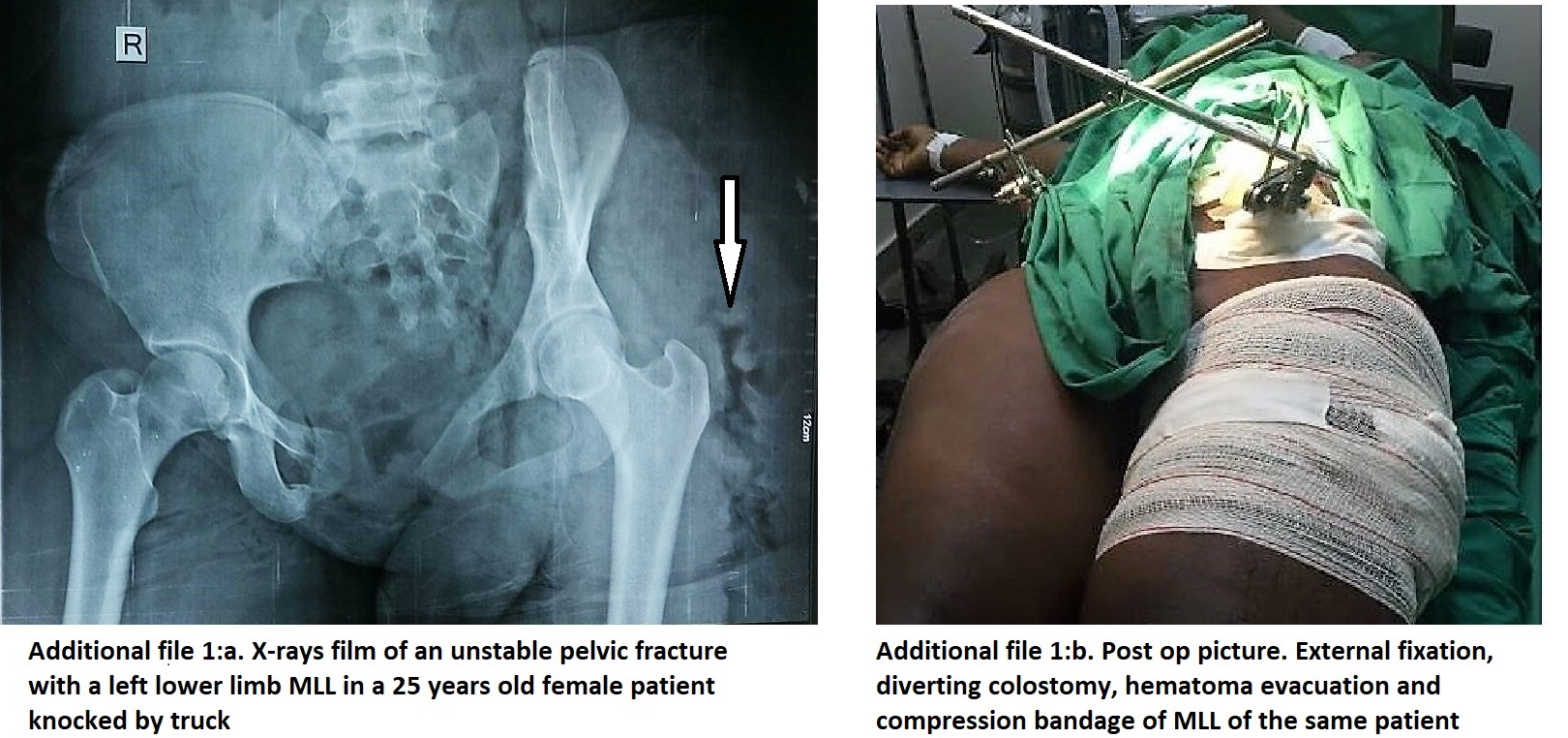

Supplement: Supplementary file 1 — Management of MLL in a 25 years old female patient knocked by a truck: a. X-rays film of an unstable pelvic fracture with a left thigh MLL; b. External fixation, diverting colostomy, hematoma evacuation and compression bandage of the MLL. (JPEG 421 kb) [file 13018_2017_706_MOESM1_ESM.jpg]

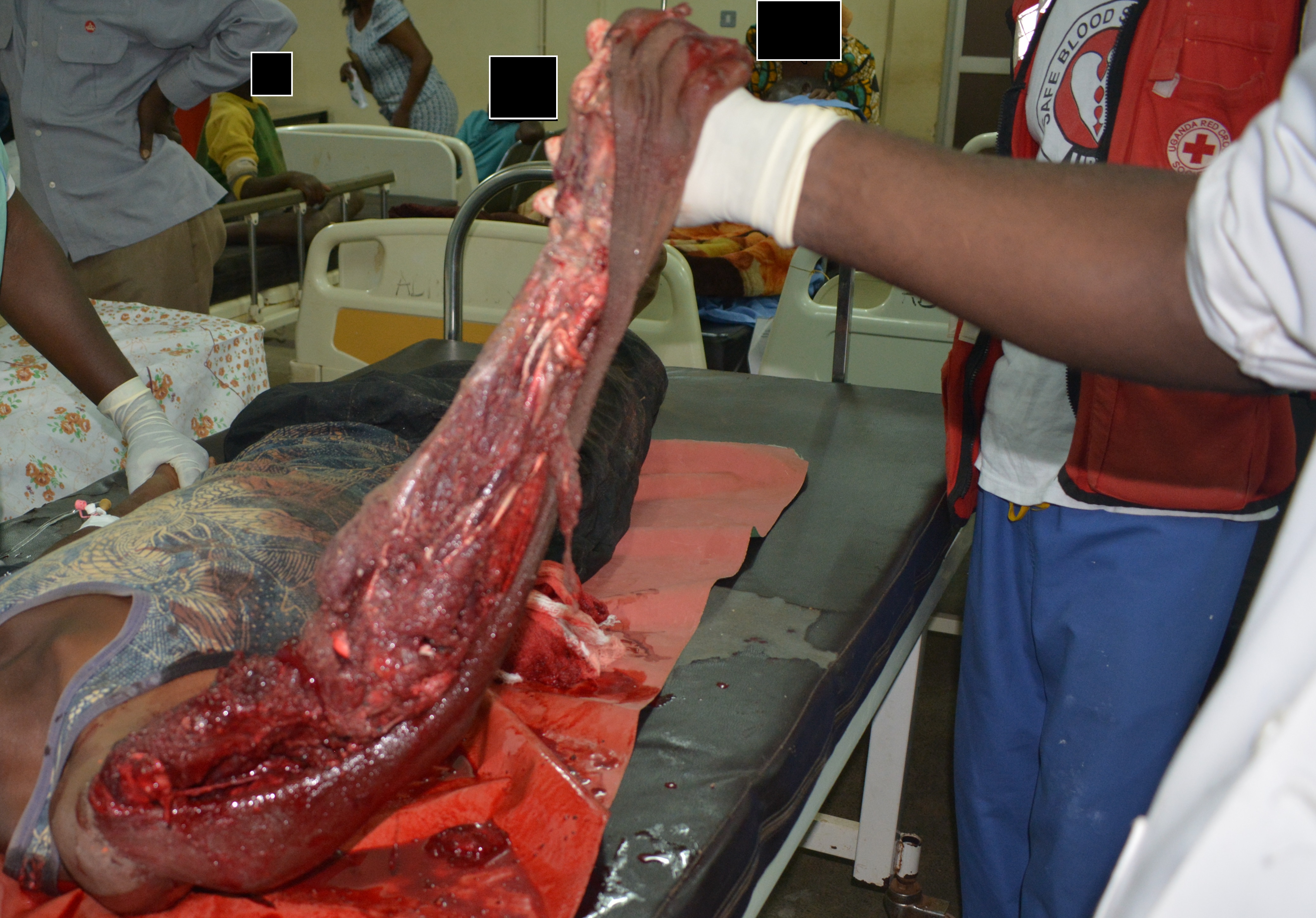

Supplement: Supplementary file 2 — Large degloving injury of the entire right upper limb of a 22 years old male patient; a construction worker who was trampled by a trailer. (JPEG 2499 kb) [file 13018_2017_706_MOESM2_ESM.jpg]

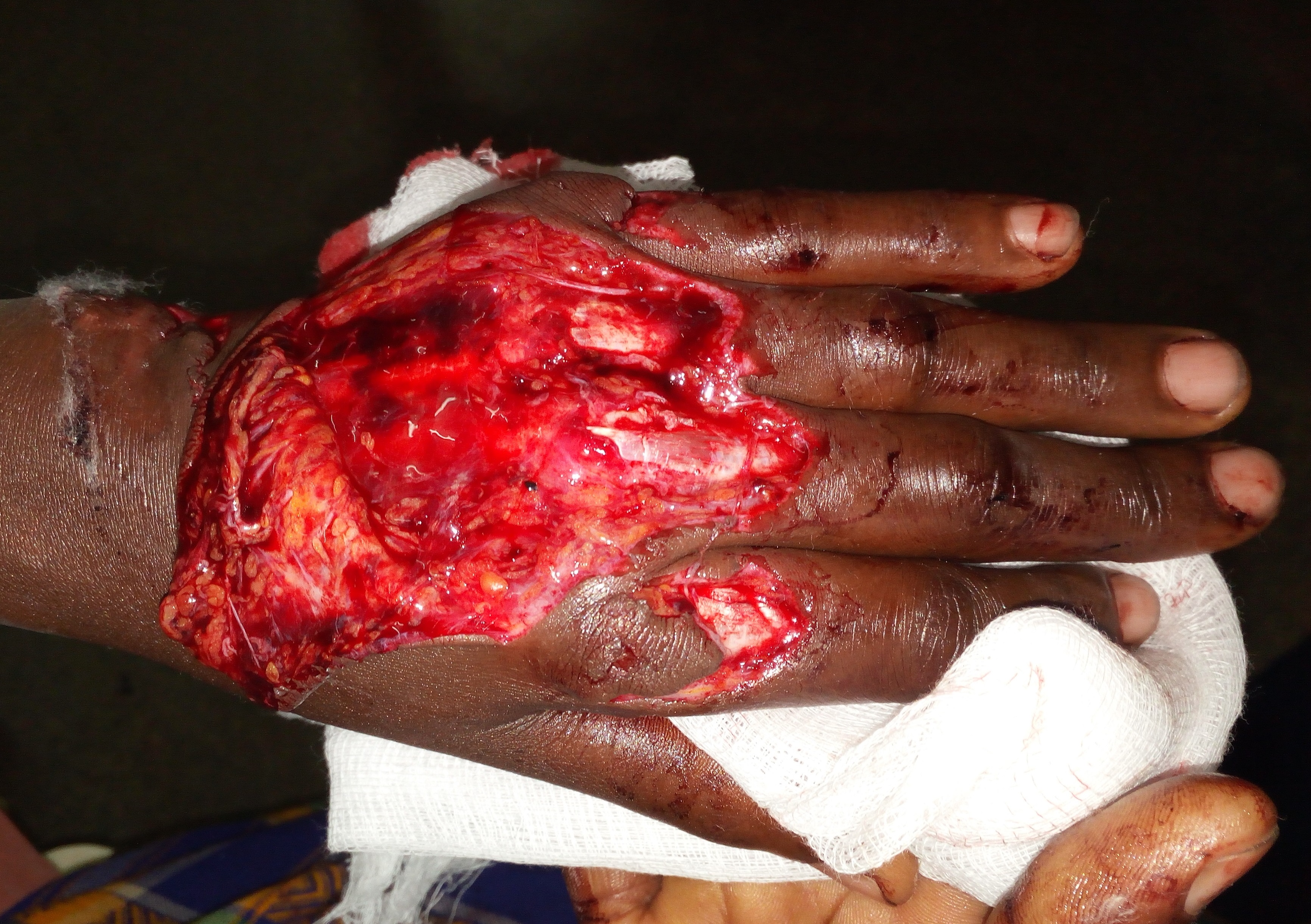

Supplement: Supplementary file 3 — Small degloving injury of the dorsum of the left hand. (JPEG 2722 kb) [file 13018_2017_706_MOESM3_ESM.jpg]

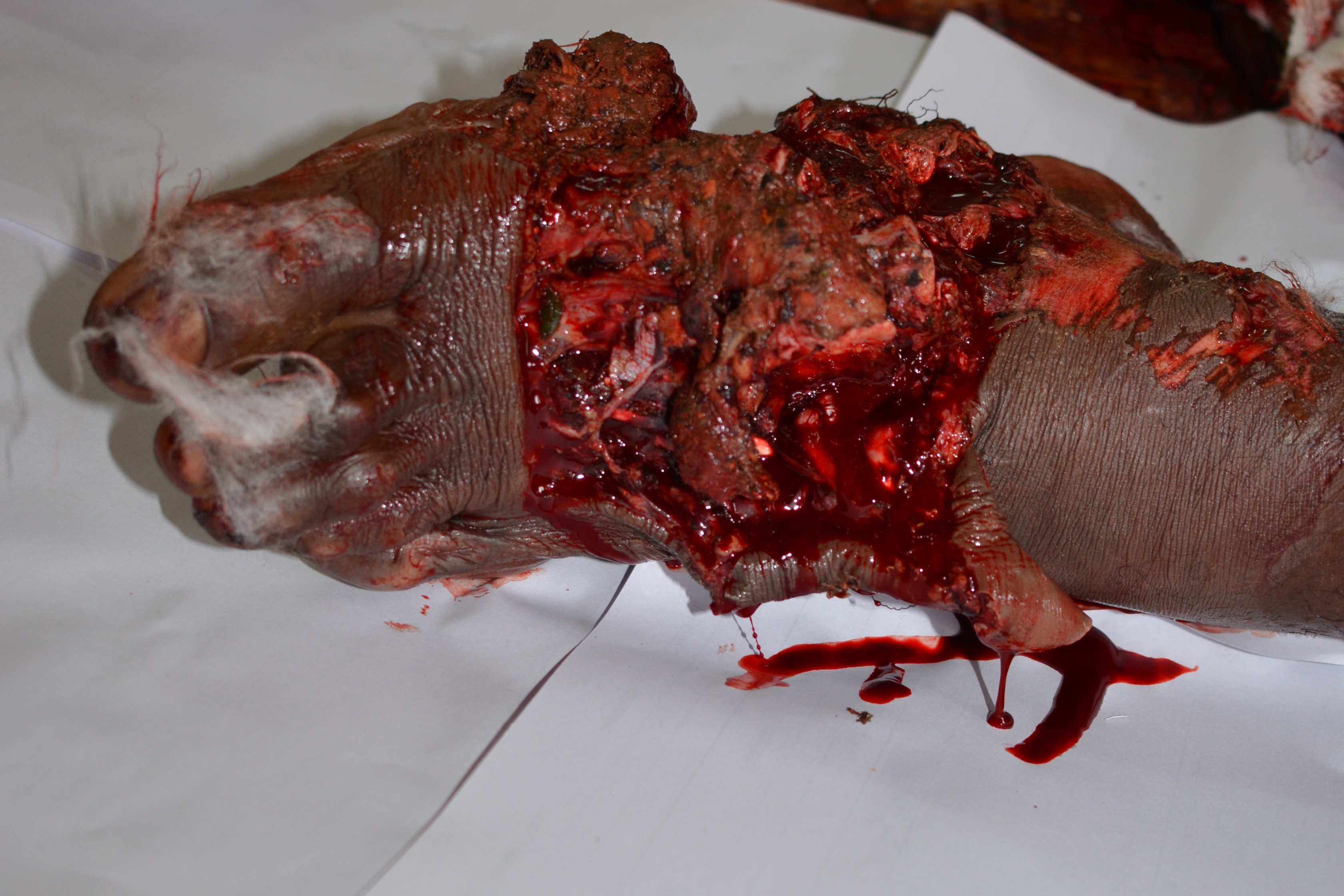

Supplement: Supplementary file 4 — Small degloving injury of the dorsum of the left foot. (JPEG 1363 kb) [file 13018_2017_706_MOESM4_ESM.jpg]
